# The detrimental effects of emotional process dysregulation on decision-making in substance dependence

**DOI:** 10.3389/fnint.2012.00101

**Published:** 2012-11-07

**Authors:** Anna Murphy, Eleanor Taylor, Rebecca Elliott

**Affiliations:** Neuroscience and Psychiatry Unit, University of ManchesterManchester, UK

**Keywords:** addiction, emotion, cognition, reward, stress, decision-making

## Abstract

Substance dependence is complex and multifactorial, with many distinct pathways involved in both the development and subsequent maintenance of addictive behaviors. Various cognitive mechanisms have been implicated, including impulsivity, compulsivity, and impaired decision-making. These mechanisms are modulated by emotional processes, resulting in increased likelihood of initial drug use, sustained substance dependence, and increased relapse during periods of abstinence. Emotional traits, such as sensation-seeking, are risk factors for substance use, and chronic drug use can result in further emotional dysregulation via effects on reward, motivation, and stress systems. We will explore theories of hyper and hypo sensitivity of the brain reward systems that may underpin motivational abnormalities and anhedonia. Disturbances in these systems contribute to the biasing of emotional processing toward cues related to drug use at the expense of natural rewards, which serves to maintain addictive behavior, via enhanced drug craving. We will additionally focus on the sensitization of the brain stress systems that result in negative affect states that continue into protracted abstinence that is may lead to compulsive drug-taking. We will explore how these emotional dysregulations impact upon decision-making controlled by goal-directed and habitual action selections systems, and, in combination with a failure of prefrontal inhibitory control, mediate maladaptive decision-making observed in substance dependent individuals such that they continue drug use in spite of negative consequences. An understanding of the emotional impacts on cognition in substance dependent individuals may guide the development of more effective therapeutic interventions.

## Introduction

Drug addiction is a persistent disorder characterized by compulsive-seeking and taking of drugs, loss of control over intake, and negative emotional states in withdrawal, such as dysphoria, anxiety, and irritability (Koob and Le Moal, 2008b). Many people try drugs; an estimated 36% of people aged between 16 and 59 have engaged in illicit drug use, the highest incidence of use being reported in young adults aged under 25 (Department of Health, 2011). For the majority, drug use is controlled, limited to a short period of time and does not result in problems. However, a small proportion develop substance dependence which is defined in the Diagnostic and Statistical Manual of Mental Disorders (DSM) as excessive drug use that may result in tolerance and withdrawal symptoms, an inability to cut down on drug use, and continued drug use in spite of knowledge of negative consequences. The term “addiction” is used by the National Institute of Drug Abuse (NIDA) to describe a chronic, relapsing disorder characterized by compulsive drug use in spite of harmful consequences, and roughly corresponds to the DSM definition of dependence. Substance abuse is defined in DSM as recurrent use of a substance resulting in occupational, legal, social, and interpersonal problems.

Dependence is a major medical, social, and economic problem for many countries worldwide. For example, tobacco contributes to 8.8% of deaths worldwide, alcohol to 3.2%, and illicit drugs to 0.4% (WHO, 2008). In England alone, around 24% of adult men and 13% of adult women consume hazardous amounts of alcohol, costing the economy approximately £20 billion (NHS, 2009). In 2003/2004 class A drug use cost the UK roughly £15.4 billion (Singleton et al., 2006), 90% of this cost due to drug-related crime, with the health care costs amounting to approximately £1.4 billion per year (Lingford-Hughes et al., 2010). While an extensive range of drugs are abused, opiates, cocaine, and alcohol have been identified as the three drugs most dangerous to both the individual and society (Nutt et al., 2010) and they will be the focus of this review.

Drug dependence is associated with changes to brain structural, neuropsychological, and emotion systems (Asensio et al., 2010). These changes have the potential to influence vulnerability for substance dependence, contribute to the maintenance of problem drug use once it has started, as well as affecting the likelihood of relapse following detoxification. Clinically and therapeutically it is important to understand the mechanisms of each of these three stages of addiction. Identification of vulnerability markers for problem drug use would allow the possibility of early intervention, or even preventative therapies in high-risk individuals. Understanding the mechanisms of maintenance of drug-taking behavior is important for preventing initial drug use from developing into dependence. Perhaps the most difficult problem facing the treatment of addiction is the very high rate of relapse following initially successful treatment (Sinha, 2011), and it is therefore crucial to understand the factors involved, in order to break the cycle of repeated detoxification and relapse.

It is clear that substance dependence is a multi-factorial problem, with a range of social, environmental, cognitive, and neurobiological factors contributing to vulnerability, maintenance, and relapse. The focus of this special issue is the interaction between cognition and emotion, and we therefore focus on this aspect of addiction research.

We will argue that substance dependence is a disorder characterized by dysregulation of emotional processes with a particular focus on reward circuitry, involved in motivation and reinforcement, and stress circuitry involved in defense. Reward and stress processing includes the modulation of cognitive performance by the presence (or absence) of motivationally salient outcomes, while stress responsivity additionally incorporates a component of achieving, or maintaining, successful cognitive performance under conditions of emotional stress and anxiety. Both these aspects of cognitive-emotional interaction are dysfunctional in individuals dependent on drugs, and we will describe how these dysfunctions may result in maladaptive behaviors that both initiate and maintain dependence and increase the risk of relapse during a period of abstinence. Specifically, we will consider how emotional dysregulation may contribute to cognitive impairments in the domains of impulsivity and decision-making, aspects of processing which may contribute to the development and maintenance of drug misuse.

### What are emotions?

The term emotion has been applied to a diverse array of perceptions, behaviors, and psychological states (Cardinal et al., 2002). We adopt the definition of emotion recently put forward by LeDoux—that emotions are phenomena that reflect functions of circuits allowing an organism to survive and thrive by detecting and responding to salient challenges and opportunities within the environment. That is, emotions are brain “responses that occur when in danger, or when in the presence of a potential mate, or in the presence of food when hungry or drink when thirsty …..” (LeDoux, 2012). By “emotional processing” we refer to the processing of information within these circuits. This operationalized definition removes the focus away from emotions reflecting subjective feeling states (which present problems when assessing emotions in animals) toward emotions reflecting processes that are experimentally tractable (LeDoux, 2000).

Emotional circuits detect key trigger stimuli (or unconditioned stimuli) on the basis of innate, hard-wired programming that has evolved through natural selection (LeDoux, 2012). These unconditioned stimuli can be potential sources of immediate pleasure, threat, or satisfaction of homeostatic need, such as immediate stress or withdrawal relief (Verdejo-Garcia and Bechara, 2009). Activation of emotional circuitry has a number of consequences within the brain and body. Changes include increased release of neurotransmitters such as dopamine, noradrenaline, and serotonin within the brain, and changes within the internal milieu and viscera of the body such as release of hormones and increased heart rate (Bechara and Damasio, 2005). In addition, emotional circuit activation can result in the conscious feeling states that are commonly associated with the word “emotion.” Emotion circuit activation also results in hard-wired, innate behavioral responses such as approach, freezing, and fleeing (van der Meer et al., 2012).

### Pavlovian conditioning

When emotional circuits are activated, learning occurs, with the establishment of an association between innate triggers or biologically significant events (referred to as unconditioned stimuli, UCS) and previously neutral stimuli that occurred in close association with them (Rescorla, 1988). The previously neutral stimuli acquires motivational value, reflecting the utility or value of the UCS (Seymour and Dolan, 2008), and acquires the ability to activate emotional circuitry themselves, thereby becoming conditioned stimuli (CS) (LeDoux, 2012). This emotional learning process is referred to as Pavlovian conditioning, after Ivan Pavlov, the discoverer of this phenomenon (Cardinal et al., 2002). This ability of the CS to predict the value or utility of the UCS results in an *expectancy* of the UCS upon presentation of the CS (Seymour and Dolan, 2008), which enables appropriate responses to be evoked by the CS in anticipation of the UCS. The amygdala is the brain structure that is considered to have a central role in Pavlovian conditioning, as well as a crucial role in emotional circuitry involved in processing reward and threat (Cardinal et al., 2002; LeDoux, 2007).

## Pathways into substance dependence

### Emotional personality traits and the risk for substance use and dependence

Motivation to engage in substance use, in addition to numerous psychosocial factors, has been related to the ability of a substance to produce positive emotional states (Volkow et al., 2011). This is also referred to as positive reinforcement. A desire for substance induced pleasure appears to be associated with certain personality traits. The trait of sensation-seeking is defined by the need for novel, varied, and intense experiences (Zuckerman, 1994) and, as will be reviewed later, is associated with functioning within the reward circuitry. Higher levels of sensation-seeking are found in alcohol-dependent individuals (Noel et al., 2011) and in young adults with alcohol use disorders (Shin et al., 2012). Higher levels of sensation-seeking have also been reported in cocaine-dependent individuals (Patkar et al., 2004; Ersche et al., 2010b) and shown to be negatively associated with treatment outcomes (Patkar et al., 2004). Mixed results have been found in opiate-dependent individuals, with higher levels of sensation-seeking found in some studies (Le Bon et al., 2004; Lemenager et al., 2011) but not others (Nielsen et al., 2012). The trait has also been shown to be associated with early alcohol use in adolescents (Martin et al., 2002; Gillespie et al., 2012; Nees et al., 2012) and is predictive of the later development of alcohol abuse (Cloninger et al., 1988) and the frequency and quantity of alcohol and polysubstance use in young adults (Chakroun et al., 2004; Woicik et al., 2009).

Motivations for drug and alcohol use in sensation seekers are associated with the enhancement of positive affect states (Comeau et al., 2001; Woicik et al., 2009). By contrast, another motive for engaging in substance use is to reduce negative affect states (Koob and Le Moal, 2008a), also referred to as negative reinforcement. Thus, a tendency to experience more negative states could increase the risk of developing substance dependence. In line with this hypothesis, high self-report measures of anxiety sensitivity are related to anxiolytic and opiate drug use in young adults, problem drinking in adolescents (Woicik et al., 2009), and early alcohol initiation in adolescents (Kaplow et al., 2001). Furthermore, the characteristic of “hopelessness,” which closely relates to depressive personality traits, is associated with a higher degree of sedative drug use in young adults as well as quantity and frequency of alcohol use in adolescents (Woicik et al., 2009). Motivation for substance use in those with anxiety sensitive and depressive traits is associated with relieving these negative affect states (Comeau et al., 2001; Woicik et al., 2009). It is important to note that other studies in young adults have failed to find an association between increased negative affect states and drug and alcohol use (Chakroun et al., 2004; Gillespie et al., 2012). These discrepancies suggest that there is considerable variability, consistent with the hypothesis of multiple routes into drug and alcohol dependence. It is likely that different personality traits will confer vulnerability via interactions with different environmental triggers, and therefore studies in different populations may show discrepant results.

Another important issue is whether emotional personality traits confer differential risk for specific substance dependences, and there is some evidence suggesting this to be the case. Studies suggest that traits of anxiety sensitivity and hopelessness are more related to anxiolytic and opiate dependence respectively, while sensation-seeking and impulsivity confer a greater risk of alcohol and cocaine dependence respectively (Conrod et al., 2000). High scores on anxiety sensitivity are more associated with primary use of heroin compared to cocaine, or the use of both heroin and cocaine (Lejuez et al., 2006) and high scorers on anxiety sensitivity are less likely to identify cocaine as their drug of choice compared to those with moderate anxiety sensitivity (Norton et al., 1997). These preferences may reflect the anxiolytic and the anxiogenic effects of opiates (Lejuez et al., 2006; Colasanti et al., 2011) and cocaine (Yang et al., 1992) respectively.

However, once again the evidence is far from conclusive. While Conrod et al. (2000) observed no association between negative affect personality traits and alcohol dependence, Norton et al. demonstrated that high scorers on traits of anxiety sensitivity indicated alcohol to be their drug of choice (Norton et al., 1997). Carpenter and Hasin demonstrated that in a sample of heavy drinkers the tendency to drink in an attempt to cope with negative affect was a risk factor for the subsequent development of alcohol dependence (Carpenter and Hasin, 1998). Furthermore, longitudinal studies have shown that adolescents with symptoms of depression and anxiety were at a greater risk of developing alcohol dependence (Mackie et al., 2011; McKenzie et al., 2011). Such findings may reflect the ability of alcohol to reduce negative affect (Gilman et al., 2008). Negative affect has also been associated with cocaine use, with higher levels of associated with cocaine use in a community-based sample of young adults (Kilbey et al., 1992) and depression in adolescents predicted higher cocaine use the following year (Newcomb and Bentler, 1986). These findings are contrary to the hypothesis that negative affect traits selectively confer enhanced risk of opiate and anxiolytic abuse. Studies have shown that major depressive disorder is prevalent in cocaine-dependents and the presence of depression may impact upon the severity of addiction (Rounsaville, 2004), further supporting an association between negative affect traits and cocaine dependence. However, longitudinal studies assessing the relationship between these traits and the subsequent development of cocaine dependence are required to determine the degree to which negative affect can be considered a cause, and/or a consequence of cocaine dependence.

While questions remain about the relationship between traits and specific addictions, it is clear that in general, affective personality traits can modulate drug use, resulting in early, and more frequent substance use and substance dependence. Sensation-seeking appears to be related to enhancing positive affect states and may be associated with earlier, heavier, and more frequent substance use in young adults, and particularly with alcohol and cocaine dependence. By contrast, it appears that personality traits associated with negative affect are more likely to be associated with use of sedatives and opiates. However, this is by no means a complete dissociation. Some studies have indicated that sensation-seeking may be elevated in heroin users. Similarly, negative affect may play a role in cocaine and alcohol dependence, particularly the transition from heavy use to dependence.

### Neurobiology underlying personality risk factors for substance dependence

These personality risk factors for substance dependence are assumed to exert their influence via altered functioning of brain motivational systems, leading to differential susceptibility to seek out specific drug-reinforcement effects (Conrod et al., 2000). In this section of the review, we will outline the underlying neurobiology of traits of sensation-seeking, hopelessness, and anxiety/stress sensitivity and how these neurobiological markers may lead to the development and maintenance of substance dependence.

#### Sensation-seeking and reward neurobiology

Rewards have been defined as hedonic incentives that cause neural representations that elicit motivation and goal pursuit, and as stimuli that positively reinforce behavioral acts (Kelley and Berridge, 2002; Schultz, 2006), thus the term “reinforcer” is often used interchangeably with “reward.” The processing of rewards is complex, involving different psychological components including “liking” or hedonic impact of rewards, “wanting” or motivation for rewards, and learning, the formation of associations through experience that allow for predictions of future rewards (Berridge and Kringelbach, 2008). Stimuli associated with a reward acquire the ability to elicit innate emotional responses that are normally associated with the reward itself, via Pavlovian conditioning.

Sensation-seeking is thought to reflect the function of an underlying motivational system or behavioral approach system (Gray, 1990) that is activated by reward signals, and represents a heightened sensitivity to these signals (Depue and Collins, 1999). Specifically, reward signals can elicit a motivational state referred to as “positive incentive motivation” which serves to guide approach behavior toward a goal. Positive incentive motivation is associated with strong positive affect such as desire, excitement, enthusiasm, energy, or self-confidence (Depue and Collins, 1999). Key brain areas involved with the processing of rewards include ventral striatum, orbitofrontal cortex (OFC), ventral pallidum, anterior cingulate cortex, and midbrain dopamine neurons (Haber and Knutson, 2010). Functional magnetic resonance imaging (fMRI) studies have demonstrated a positive correlation between blood oxygen level dependent (BOLD) response in these key reward processing areas and measures of reward sensitivity (Beaver et al., 2006; Hahn et al., 2009) and self-reports of excitement in response to reward cues (Bjork et al., 2004b, 2008c). A positive correlation has been reported between ventral striatal activation and trait measures of sensation-seeking (Bjork et al., 2008a), supporting the theory that sensation-seeking reflects enhanced reward sensitivity.

***Dopamine and reward sensitivity.*** Seminal theories posit that ventral tegmental dopamine release into the ventral striatum mediates reward sensitivity by encoding the intensity or “salience” of reward related stimuli (Robinson and Berridge, 1993), or the predictive value of conditioned reward stimuli and the error in the prediction of unconditioned stimuli whenever they are surprising (Schultz, 1998). Therefore, dopamine may influence the motivational value of stimuli and their impact on emotional and behavioral responses (Depue and Collins, 1999).

In support of this theory, ventral striatal response to reward cues, measured by fMRI, has been shown to correlate with ventral striatal dopamine release, measured by positron emission tomography (PET) (Schott et al., 2008). Dopamine-enhanced incentive salience of stimuli has been suggested to increase incentive motivational states and make stimuli, or their associated reward, more attractive or “wanted.” Dopamine-mediated enhancement of “wanting” has been suggested to underlie the craving that is often experienced by drug users after exposure to drug related stimuli (Robinson and Berridge, 1993).

***Dopamine and sensation-seeking.*** Sensation-seeking is assumed to reflect heightened reward sensitivity, which is hypothesized to be modulated by dopamine. This may suggest that sensation-seeking could also be influenced by dopamine. Genetic linkage studies demonstrate associations between sensation-seeking and polymorphisms of dopamine-related genes (Zuckerman, 2005; Golimbet et al., 2007; Munafo et al., 2008) and a recent PET study reported an inverted “U” shaped relationship between striatal dopamine D2/D3 receptor availability and scores of sensation-seeking (Gjedde et al., 2010). Such a relationship indicates that dopamine receptor availability rises with sensation-seeking at lower scores, but falls in opposition to sensation-seeking scores at the higher end. The authors propose that high levels of sensation-seeking reflects a hyperdopaminergic state, that results in reduced D2/D3 availability for the competing PET radioligand, as opposed to a reduced D2/D3 receptor density in these individuals (Gjedde et al., 2010). This is supported by studies indicating higher sensation-seeking to be associated with reduced activity of monoamine oxidase, resulting in higher brain dopamine concentrations (Zuckerman, 1985; Golimbet et al., 2007). Furthermore the closely related trait of novelty-seeking is found to be inversely correlated with midbrain D2/D3 autoreceptor availability, with higher novelty-seeking related to lower autoreceptor availability (Zald et al., 2008). In contrast, to post-synaptic dopamine receptors, midbrain dopamine receptor availability remains relatively constant after pharmacological manipulations that alter dopamine levels. Therefore, receptor availabilities of midbrain autoreceptors found in this study are assumed to reflect receptor densities, rather than levels of competing endogenous dopamine (Zald et al., 2008). The DA autoreceptor exerts a powerful inhibitory effect on dopamine neuron firing (Aghajanian and Bunney, 1977), thus lower autoreceptor densities found in high novelty seekers might be expected to result in higher levels of dopamine release. This hypothesis is supported by studies demonstrating that novelty-seeking positively correlates with drug-induced dopamine release (Leyton et al., 2002; Boileau et al., 2006). In addition to the role of dopamine in sensation/novelty-seeking, other neurotransmitter systems may also be involved as interactive effects between dopamine- and serotonin-related genes and novelty-seeking have been reported (Zuckerman, 2005).

#### Negative affect traits and reward neurobiology

***Depressive personality traits.*** Two core features of depression are a markedly reduced interest or pleasure in activities and low mood (feelings of sadness), thus depressive personality traits are also linked to dysfunction of brain reward and motivational systems, and may specifically relate to hypofunctioning of the mesolimbic dopaminergic system (Pizzagalli et al., 2009). Most neurobiological theories of depression focus on the serotonergic and noradrenergic systems, since all effective anti-depressant medications converge upon these systems. However, preclinical evidence suggests some of the therapeutic effects of anti-depressants may be partly due to increased striatal dopamine transmission and enhanced sensitivity within the mesolimbic dopamine system (Markou et al., 1998). Furthermore, recent studies demonstrate that manipulations of proteins regulating ventral tegmental dopamine produce behavioral phenotypes relevant to depression (Nestler and Carlezon, 2006).

***Anxiety sensitive personality traits.*** Anxiety sensitivity is thought to reflect functions of a defence system that is activated by aversive, novel, and innate fear stimuli (Barros-Loscertales et al., 2006). Anxiety is a motivational state that promotes adaptive behaviors; it is, however, distressing for the organism and impairs performance when excessive (Colasanti et al., 2011). The anxiety/stress system is modulated by numerous neurotransmitters including corticotrophin releasing factor, neuropeptide Y, substance P, noradrenaline, serotonin, dopamine, glutamate, and GABA—see Charney and Drevets (2002) for detailed review. In contrast to sensation-seeking and depressive personality traits, anxiety sensitivity may not directly reflect abnormalities within the brain's reward system but instead may reflect indirect effects. For example, it has been suggested that concomitant inputs from key anxiety structures affect the way neural signals are “gated” within the nucleus accumbens such that they not rewarding but instead serve to increase motivation to deal with the threat at hand (Nestler and Carlezon, 2006).

## Reward sensitivity theories of substance dependence

These findings raise the crucial question of how sensation-seeking, depressive, and anxiety sensitive personality traits confer increased risk of developing addiction. There is evidence that sensation-seeking and depressive traits reflect dopaminergic disturbances which influence response to reward and control of incentive motivation. We have presented evidence that these premorbid traits result in early, more frequent use of substances. In the following sections we will also present evidence of additional drug-induced adaptations within the reward circuitry that are proposed to enhance the desire to engage in substance use, contributing to the subsequent development of substance dependence.

Dysfunction within brain reward systems, where dopamine signaling has important functions, has been widely studied in addiction. Alterations in reward responsiveness and incentive motivation represent an important way in which emotional processing can impact cognitive function, resulting in poorly controlled, and maladaptive behavior. In this section, we will briefly outline two major theories of impaired reward sensitivity in addiction.

### Incentive sensitization in substance dependence

Sensation-seeking is assumed to reflect heightened reward sensitivity and heightened positive incentive motivation. Sensation seekers may have enhanced motivation to engage in initial substance to further increase positive affect states. The transition from controlled recreational drug use to compulsive use is hypothesized to be the result of drug-induced sensitization of mesocorticolimbic brain systems that attribute incentive salience to reward-associated cues. The main points of this theory are that previously neutral stimuli acquire incentive motivational properties through association with drug rewards via Pavlovian conditioning mechanisms. Therefore, exposure to conditioned drug cues can produce dopamine release from mesolimbic dopamine neurons that causes drug wanting. Repeated exposure to addictive substances may result in neuroadaptation in mesolimbic dopamine neurons that sensitize these neurons (Robinson and Berridge, 1993). This effect has been demonstrated in animal models as an enhanced expression of psychomotor activating effects of all drugs of abuse, which is thought to be dependent upon the mesolimbic dopaminergic system (Robinson and Berridge, 2001). In humans, enhanced release of dopamine after a repeated dose of amphetamine has been observed (Boileau et al., 2006) and enhanced presynaptic dopamine function has recently been reported in ex-recreational users of psychostimulant drugs, although it is unknown whether this reflects a pre-existing hyperactivity or a drug induced effect (Tai et al., 2011). This sensitization of mesolimbic neurons is suggested to result in pathological levels of incentive salience being attributed to drugs and drug cues, thus creating a pathological incentive motivation for drugs which can persist for years (Robinson and Berridge, 2008).

### Reward deficiency syndrome

Dopamine is related to incentive motivational aspects of rewards which are in turn associated with strong positive affect such as excitement, enthusiasm, and self-confidence (Depue and Collins, 1999). Many drugs of abuse, either directly or indirectly, induce acute release of dopamine from mesolimbic dopamine neurons into the nucleus accumbens in rodents (Di Chiara et al., 2004), while stimulants but not heroin, have been demonstrated to increase dopamine release into the ventral striatum in humans (Lingford-Hughes et al., 2010) with mixed findings with respect to alcohol and dopamine release in humans (Boileau et al., 2003; Yoder et al., 2009). Pleasurable feelings of intoxication correlate with ventral striatal dopamine release for stimulants (Volkow et al., 1999) and alcohol (Boileau et al., 2003) and thus dopamine may be important for the rewarding effects of drugs of abuse (Volkow et al., 2011). However, the idea that drug-induced dopamine release mediates the hedonic impact of drugs of abuse is controversial (Wachtel et al., 2002; Berridge, 2007). Instead these rewarding effects may occur via the enhancement of the perceptual impact or incentive salience of environmental stimuli (Everitt and Robbins, 2005).

The opioid system is related to consummatory aspects of reward such as satiation, sedation, and “bliss” (Comings and Blum, 2000). Generally speaking the pleasurable feelings associated with opiate drugs are due to mu and delta opioid receptor agonism (Le Merrer et al., 2009). Heroin exerts its euphoric effects through mu opioid receptor agonism, as blockade of these receptors has been demonstrated to reduce opiate self-administration (De Vries and Shippenberg, 2002). However, emerging evidence suggests the opioid system is not only involved in the reinforcing effects of heroin, but also those of alcohol (Mitchell et al., 2012) and amphetamine (Jayaram-Lindstrom et al., 2008; Colasanti et al., 2012) via the release of endogenous opioids. Furthermore, increased mu opioid receptor binding has been found in cocaine users, suggesting an important role of the endogenous opioid system in cocaine dependence (Ghitza et al., 2010).

Comings and Blum put forward the “Reward Deficiency Hypothesis” (RDS) as one possible vulnerability for the development of substance dependence. This theory highlights the role of pre-morbid trait vulnerabilities in the subsequent development of substance dependence. According to this hypothesis, individuals with deficient reward-signaling systems may be at greater risk of developing substance dependence. In such individuals, natural rewards do not adequately stimulate the reward system, which may contribute to depressive traits associated with substance use. Therefore, it is proposed that such individuals use substances in order to enhance stimulation in deficient reward pathways.

After the development of substance dependence, the influence of negative affect becomes more apparent, suggesting that chronic drug use may lead to changes to the brain's reward system. Therefore, in addition to trait vulnerabilities in the reward system, drug-induced neurobiological changes may result in additional deficiencies in reward sensitivity. Koob and colleagues have argued that these homeostatic or “opponent” processes occur to reduce the rewarding effects of drugs of abuse (Koob and Le Moal, 2005). In support of this theory, increased tolerance to the rewarding effects of cocaine (Kenny et al., 2003), opiates (Liu and Schulteis, 2004), and alcohol (Schulteis and Liu, 2006) occurs in rodents as demonstrated in intracranial self-stimulation experiments. Acute withdrawal is associated with reduced mesolimbic dopamine release (Koob and Le Moal, 2005). Therefore, these changes are likely to underlie anhedonia and amotivation associated with withdrawal from drugs of abuse.

### Human studies assessing reward functioning in addiction

There are thus two distinct theories about how reward sensitivity may be abnormal in addiction. One theory is that substance dependence is characterized by ***enhanced*** sensitivity to reward and therefore enhanced incentive motivation toward drug and even non-drug cues (Hommer et al., 2011). However, drug use is also associated with negative affect states and the reward deficiency syndrome posits that ***reduced*** functioning of the brain's reward system underlies the motivation to engage in substance use in order to normalize these deficiencies. These theories make different predictions about how the brain reward systems will respond to a range of reward cues. Functional imaging techniques provide a means to investigate these predictions in human subjects.

#### Brain response to monetary cues

Studies have examined brain response to monetary reward using the monetary incentive delay task (Knutson et al., 2001). In alcohol-dependent individuals, there have been mixed findings. Two studies have found ventral striatal activation to be decreased in dependent individuals to controls, and negatively correlated with craving levels (Wrase et al., 2007; Beck et al., 2009). These findings therefore support the reward deficiency theory of addiction. However, another study found no difference between controls and alcohol-dependent individuals in response to monetary cues (Bjork et al., 2008c), but enhanced ventral striatal activation in response to reward outcome, a finding more consistent with the hypersensitivity hypothesis of addiction. The authors suggested that decreased ventral striatal activations for monetary cues found by Beck et al. and Wrase et al. may have been due to faster trial presentation putting too high a demand on attentional processing, rather than reduced reward sensitivity. However, a later study by the same group failed to replicate the finding of enhanced ventral striatal activation to reward outcome (Bjork et al., 2011). One possible source of the discrepant findings may be that the studies of Bjork and colleagues, in contrast to the studies of Beck et al. and Wrase et al., included alcohol-dependent participants reporting current or past substance dependence, most significantly, cocaine. Therefore, the evidence suggests that dependence upon alcohol only is associated with a reward system insensitivity. However, it is also important to note that studies in individuals already dependent on alcohol do not provide clear evidence for how that dependence developed initially. The lack of conclusive findings may reflect heterogeneity within the alcohol-dependent groups, consistent with the results of personality studies reviewed earlier. Both sensation-seeking (Conrod et al., 2000) and negative affect (Carpenter and Hasin, 1998) traits have been found to be associated with alcohol dependence. It is possible the discrepant reward sensitivity theories illustrate distinct routes into alcohol dependence for individuals with different personality traits.

Only one study to date has carried out the monetary incentive delay task in cocaine-dependent individuals, reporting no differences in ventral striatal responses between controls and cocaine-dependent individuals during reward anticipation, but enhanced ventral striatal response in cocaine-dependents for reward outcomes (Jia et al., 2011). BOLD responses during reward anticipation and outcome were found to be negatively correlated with abstinence measures and treatment retention. This finding, in addition to the enhanced vs. response in alcohol-dependent participants reporting significant cocaine dependence, suggests an enhanced reward sensitivity occurs in cocaine addiction.

Two other studies investigated brain response to monetary rewards in cocaine users with a related task, reporting no differences in ventral striatal BOLD response but disturbed OFC responsivity to different monetary value conditions within cocaine-dependent individuals (Goldstein et al., 2007a,b). Whilst OFC metabolism has been shown to depend on striatal dopamine receptor density (Volkow et al., 1993), it is difficult to draw conclusions regarding the direction of the sensitivity of mesocorticolimbic system based on these findings.

At the time of writing, we are not aware of any published brain imaging studies investigating response to monetary reward in opiate addiction. However, given that opiate addiction appears to be more related to depressive personality traits that are characterized by anhedonia and reduced motivation, that are considered to be related to deficient reward system functioning, it could be predicted that a reduced ventral striatal activation would be found to monetary cues and reward outcome in opiate addiction.

#### Brain response to drug cues

Assessing the brain response to conditioned drug-related stimuli or drug “cues” has been central to addiction research. Neural and psychological responses to drug cues are considered to be important in the maintenance of addiction and have been implicated in triggering relapse to drug use during periods of abstinence (Everitt et al., 2001).

Due to the huge number of studies assessing the brain response to drug stimuli, a comprehensive review of cue induced craving studies is beyond the scope of the current review. However, two recent activation likelihood estimation (ALE) meta-analyses of these studies have been conducted (Chase et al., 2011; Kuhn and Gallinat, 2011). Kuhn and Gallinat reported that enhanced brain response to drug cues compared to non-drug cues in alcohol and cocaine addiction converge upon the ventral striatum. Additionally, Chase et al. found areas of convergence in the ventral striatum, OFC, and amygdala in response to alcohol, heroin, and cocaine cues compared to control cues. Furthermore, ALE meta-analyses were carried out on all of the studies reporting correlations between brain response and self-reported craving. Kuhn and Gallinat found that activity within anterior cingulate cortex, ventral striatum, and pallidum correlated with craving in alcohol studies, whereas the study of Chase et al. which included a wider range of studies, found amygdala correlations.

In summary, these results suggest that drug cues, compared to non-drug cues, result in increased brain activation in key reward processing areas, and greater activation in these regions is associated with subjective craving. Reward system activation to drug cues that results in increased drug wanting supports the incentive sensitization view of addiction. However, such findings seemingly contradict reports of reduced reward system activation to monetary cues in alcohol-dependent individuals, a finding more in keeping with the reward deficiency theory. It is possible that the reward system may be overactive specifically in response to drug cues, but not other reward cues (where it may be underactive), in line with theories suggesting a biasing of reward systems toward drug-related stimuli. To explore this idea further, we will review studies of responses to natural reward cues in drug dependence.

#### Drug cues vs. natural rewards

An alternative method of probing reward functioning is to examine brain response to natural reward stimuli, that is cues that have survival significance, such as cues for food, water, and sex. Garavan et al. compared the brain response to drug films and erotic films in cocaine users and healthy volunteers. Both films activated a similar network including medial and dorsal prefrontal, parietal, cingulate and insular cortices, and subcortical regions including caudate and thalamus in drug users. Between group comparisons revealed enhanced responses in anterior cingulate, inferior parietal lobe, and caudate in drug users compared with healthy controls for the drug video, but reduced responses to the erotic video (Garavan et al., 2000). Another study investigating responses to erotic images found that cocaine users had reduced ventral and dorsal striatal and medial prefrontal responses compared to healthy controls (Asensio et al., 2010). The authors suggest this hypoactivation indicates deficient reward evaluation, motivational, and saliency attribution for natural reward stimuli.

In contrast to these studies, where natural reward stimuli and drug stimuli activated a similar network of brain regions, a study examining responses to heroin and water cues in thirsty heroin users found differential activation for different cues (Xiao et al., 2006). Water cues activated anterior cingulate cortex, whereas heroin cues activated bilateral inferior frontal cortex, cerebellum, and visual processing areas. Whilst the authors suggest that heroin and natural rewards activate different reward-related brain areas, this is not supported by earlier studies reporting anterior cingulate activation to heroin cues (Daglish et al., 2001).

Brain responses to cues for drugs and natural rewards have been measured using electroencephalography (EEG). The P300 waveform is of particular interest for the processing of stimuli, appearing 300 ms after presentation. Stimuli classified as salient attract greater attentional processing and produce larger P300s (Lubman et al., 2007).

A recent study compared subjective and electrophysiological response to images of natural reward (food, erotic) and heroin stimuli in healthy controls and heroin users. Heroin users rated natural reward stimuli as less arousing than healthy controls, and less arousing than drug stimuli. A direct comparison between P300 amplitudes for drug and natural reward stimuli indicated that amplitudes were increased for drug stimuli and reduced for natural reward stimuli in drug users (with the opposite found in controls), indicating drug cues attracted more attentional processing. Furthermore, heroin users displayed less startle-elicited P300 attenuation whilst viewing images of natural rewards relative to neutral images, compared to controls, suggesting they did not attend strongly to images of natural reward. Subjective ratings of pleasantness for the natural rewards robustly predicted later heroin use with lower pleasantness ratings associated with greater heroin use (Lubman et al., 2009). These findings of enhanced responses to drugs cues but reduced responses to natural rewards provide support for both the incentive sensitization theory of addiction and the reward deficiency hypothesis respectively, compatible with a biasing of reward systems toward drug cues and away from non-drug cues.

#### Positron emission tomography studies indexing reward sensitivity

PET has also been used to index reward sensitivity of drug users, enabling quantification of brain DA receptors by measuring radioligand binding, and indirect measures of DA neurotransmission from changes in radioligand binding. Studies examining endogenous dopamine release in response to pharmacological challenge have found that striatal dopamine release is significantly blunted in cocaine- (Volkow et al., 1997; Martinez et al., 2007), alcohol- (Martinez et al., 2005), and heroin (Martinez et al., 2012) -dependent subjects. Furthermore, the greater the reduction in dopamine release in cocaine-dependent subjects, the more cocaine was used in the treatment setting (Martinez et al., 2007), although this relationship was not found in heroin users. Radioligand D2/D3 receptor levels have consistently been found to be reduced in the striatum of cocaine- (Volkow et al., 1993; Martinez et al., 2004), alcohol- (Volkow et al., 1996; Heinz et al., 2004), and heroin (Zijlstra et al., 2008; Martinez et al., 2012)-dependent individuals, leading to the conclusion that chronic drug use is associated with reduced concentration of D2 receptors. Moreover, the reduced ventral striatal D2/D3 binding in alcohol-dependent subjects was associated with enhanced alcohol craving and enhanced prefrontal brain activation to alcohol cues, as measured with fMRI (Heinz et al., 2004).

A potential confound of these studies may be that, due to the sensitization of dopamine neurons, dopamine levels were higher at baseline in the dependent groups, resulting in reduced availability of unbound D2/D3 receptor for the competing radioligand to bind to, rather than D2/D3 density being low *per se*. However, a PET study determining baseline dopamine levels in cocaine dependence demonstrated lower levels in cocaine-dependents, indicating dopaminergic neurotransmission and D2/D3 receptors are indeed reduced in these individuals (Martinez et al., 2009).

A blunted dopamine system supports the reward deficiency hypothesis of drug dependence. However, in line with the incentive sensitization view of addiction, studies have demonstrated enhanced striatal dopamine release in response to drug cues in cocaine (Volkow et al., 2006; Wong et al., 2006) and heroin (Zijlstra et al., 2008) dependence. This dopamine release to conditioned drug cues was located to the dorsal, but not the ventral, striatum that has been implicated in habitual, stimulus-response type action selection (Everitt and Robbins, 2005; Redish et al., 2008). Cue-induced dorsal striatum dopamine release was positively correlated with acute craving levels and addiction severity in the cocaine studies and chronic craving level in the heroin study. Thus, it appears that baseline levels are low, however, in the presence of drug cues, high levels of dopamine are released that result in drug craving. This is consistent with the idea that there is an overall reward deficiency, but brain reward systems are biased to be sensitized specifically to drug cues.

#### Summary of reward system changes—the emergence of attentional bias for drug stimuli

**Figure 1:**
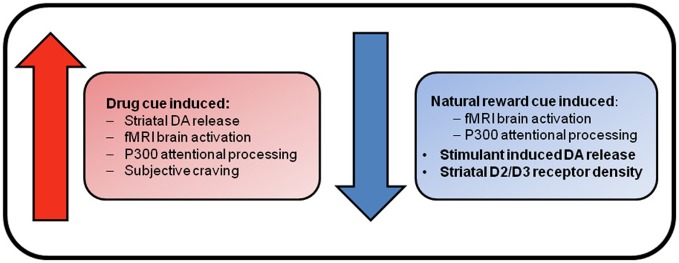
**Reward system changes associated with substance dependence.** Enhanced brain response and craving elicited by drug cues supports the incentive sensitization view of addiction. This theory suggests that repeated exposure to drugs of abuse causes neuroadaptations within mesolimbic dopamine neurons that results in pathological levels of incentive salience being attributed to drugs and their associated stimuli. In contrast, reduced brain responses for natural rewards, and blunted dopaminergic functioning in the absence of drug cues, are suggestive of deficient reward functioning. This deficient reward signaling is hypothesized to result in the seeking of drug rewards as natural rewards do not adequately stimulate the deficient reward system.

From this brief review, it is clear that there is evidence for both the incentive sensitization and reward deficiency theories of addiction (see Figure 1). The incentive sensitization theory posits that stimuli associated with drugs obtain incentive motivational properties via Pavlovian conditioning mechanisms. Repeated exposure to addictive substances results in the sensitization of mesolimbic brain circuitry that results in excessive dopamine release in response to drug cues. This is proposed to produce a heightened incentive motivation to take drugs that underlies compulsive drug-seeking in addiction (Robinson and Berridge, 1993). This theory is supported by enhanced striatal and prefrontal BOLD response to drug cues across all dependencies, and enhanced cue-induced striatal dopamine release in cocaine and heroin dependence, that was associated with drug craving, and greater attentional processing of drug cues in opiate dependence. Furthermore, enhanced brain activation to monetary reward has been found in cocaine-dependents and comorbid alcohol- and cocaine-dependent individuals (Bjork et al., 2008c; Jia et al., 2011).

The reward deficiency hypothesis argues that reduced functioning of brain reward systems underlies addiction, such that individuals seek pharmacological enhancement of their deficient reward systems because natural rewards do not adequately stimulate them. Evidence comes in the form of decreased brain response for natural reward stimuli in fMRI studies in cocaine-dependent individuals (Garavan et al., 2000; Asensio et al., 2010), decreased BOLD response to monetary reward in alcohol dependence, decreased attentional processing of naturally rewarding stimuli in opiate users as demonstrated in EEG studies (Lubman et al., 2009), and increased reward thresholds across all dependencies as demonstrated in animal studies (Koob, 2009). PET studies have also demonstrated reduced striatal D2/D3 receptor density in alcohol-dependent subjects that is associated with enhanced craving (Heinz et al., 2004), and reduced endogenous dopamine release in cocaine users that was associated with greater cocaine use (Martinez et al., 2007).

There are numerous possible explanations for the reported findings. Different findings in response to monetary cues in alcohol and cocaine dependence suggest that different substances of abuse are associated with different reward system abnormalities. Such differences may reflect differences in pre-existing trait vulnerabilities for substance dependence that are hypothesized to reflect hyper and hypo activity of reward systems such as sensation-seeking and negative affect traits respectively (that result in different motives for the engagement of substance use), or distinct pharmacological effects of the different drugs themselves on brain reward systems. However, although behavioral sensitization is most commonly demonstrated with psychostimulant drugs, it has also been demonstrated with most drugs of abuse in animals (Narendran and Martinez, 2008). Furthermore, reward sensitivity may change over the course of one's drug using career, initially reflecting pre-existing traits that predispose individuals to engage in substance use, but then being modulated by sensitization of dopamine neurons after drug use that increases the motivational salience of drug rewards. Continual drug exposure may however, result in the dominance of opponent processes that counteract sensitization and the chronic presence of drugs of abuse. This may ultimately result in an allostatic shift to deficient reward functioning, producing a dependence upon substances of abuse in order to restore reward deficits. This is suggested by the finding that recreational psychostimulant use is associated with hyperactive dopaminergic activity (Tai et al., 2011), perhaps reflecting sensation-seeking traits and/or drug induced sensitization but chronic psychostimulant use is associated with blunted dopaminergic activity, the severity of which is associated with greater drug use (Martinez et al., 2009). Although enhanced brain and attentional responses to drug cues are detected in chronic drug users, the reward deficiency hypothesis posits that drug related cues are “framed” as especially salient in comparison to non-drug rewards, due to their greater ability stimulate deficient reward systems, resulting in bias toward drug-related stimuli (Hommer et al., 2011). Therefore, perhaps it is the *contrast* between dopaminergic response to drug cues compared to natural rewards and deficient baseline activity, that is the important factor in driving drug-seeking in chronic drug users, rather than overall higher activity levels of dopamine neurons (neuroimaging studies do not measure absolute levels of dopamine release or brain activity in response to drug cues, but instead use indirect measures that involve comparisons with an unknown baseline). The ability of drugs of abuse to potently activate brain reward systems is one reason why drugs of abuse are overvalued within the brain (Redish et al., 2008). In contrast, the relative impotence of natural rewards in activating deficit reward systems may result in natural rewards being undervalued in the brain of a chronic drug user. The amygdala is crucial for emotional associative learning and generating responses to CS, specifically allowing a conditioned stimulus to access the value of the reward that it predicts. This information can be used to modulate motivation via inputs to midbrain dopamine neurons, and instrumental actions via projections to ventral striatum and prefrontal cortex (Cardinal et al., 2002). Therefore, the amygdala may be an important neural structure involved in the “framing” of salience of drug cues over natural rewards by translating differences in stored value representations between drugs and natural rewards into differential activity of brain motivational systems.

An alternative explanation for the findings that support both the incentive sensitization and the reward deficiency hypothesis may be that both hyperfunctioning and hypofunctioning brain reward systems occur simultaneously in addiction depending upon the presence or absence of conditioned cues or contexts. Neural sensitization of dopamine neurons may be influenced by associative learning mechanisms such that enhanced neural sensitization occurs for drug cues and contexts but not for non-drug contexts (Leyton, 2007; Robinson and Berridge, 2008). In animals, sensitized increases in dopamine release to cocaine occurred only when animals were tested in an environment where they had previously experienced drug, and not in an unfamiliar environment (Duvauchelle et al., 2000). This may explain why increases in dopamine were detected in dependent subjects in response to drug cues, but not in response to pharmacological challenge in a novel environment in the absence of cues. Homeostatic “opponent” processes (Koob and Le Moal, 2005) may be initiated simultaneously in response to chronic elevations in dopamine and opioid levels, such that in the absence of drug cues, the reward system is hypoactive. Given the important role of the amygdala in associative learning and the generation of responses to CS, it is likely to be key structure involved in modulating the expression of incentive sensitization by allowing the high values of drugs of abuse to influence mesolimic dopamine systems after exposure to drug cues and contexts (Volkow et al., 2010).

**Figure 2:**
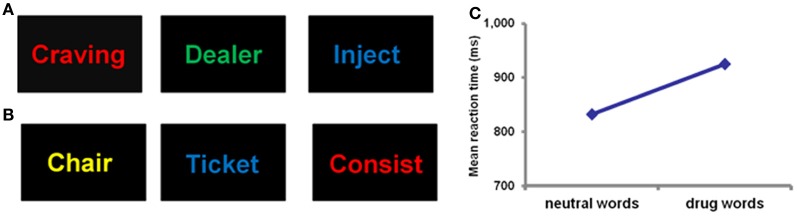
**Example of a drug word emotional Stroop task showing drug words (A) and neutral words (B).** Participants are required to identify the color of the text as quickly as possible. Successful performance of this task requires the suppression of emotional responses to word meaning, and a direction of attention toward non-emotional content (word color). Slower reaction times are assumed to indicate a greater degree of emotional interference on cognitive processing. Panel **(C)** demonstrates that heroin dependent individuals have significantly slower reaction times for drug words compared to neutral words (Murphy et al., 2011), reflecting the emotional significance of the drug words compared to neutral words.

Although the relationship between reward sensitivity and addiction is complex, it is clear that reward sensitivity is compromised, with a clear bias toward drug rewards once addiction is established. Enhanced motivational salience of drugs and related cues in addicted individuals leads to a biasing of attentional and cognitive processing toward drug-related cues (Goldstein and Volkow, 2002). This attentional bias, the automatic selective attentional response to emotionally salient stimuli, has been demonstrated in drug word Stroop (Figure 2) and dot probe detection tasks across heroin (Franken et al., 2000; Lubman et al., 2000; Marissen et al., 2006), alcohol and cocaine (Lusher et al., 2004; Hester et al., 2006; Ersche et al., 2010a) dependencies. Enhanced motivational salience of reward cues is attributed to increased dopamine release in the ventral striatum (Berridge, 2007) and enhanced attentional processing of drug cues may be mediated by this dopaminergic activity (Franken, 2003). In addition to this specific action, “general arousal” effects produced by emotional circuit activation may contribute to the biasing of attention toward emotional stimuli (LeDoux, 2012). Generally increased arousal, produced by the release of noradrenaline, serotonin, and acetylcholine as well as dopamine, facilitates processing in the emotion circuit that triggered the arousal response initially, and in sensory, cognitive, and memory systems. The overall effect is that brain systems are coordinated and monopolized for the purpose of enhancing the ability of an organism to benefit from an opportunity or cope with a challenge (LeDoux, 2012). Whilst this action of emotional circuit activation normally serves to benefit an organism, this emotional influence over cognitive systems in substance dependence leads to maladaptive behavior. Unlike natural rewards such as food and sex, drugs of abuse do not have survival significance, but instead they simulate the action of natural rewards on the brain (Kelley and Berridge, 2002; Redish et al., 2008). Biasing attention toward stimuli of substances that are not beneficial for survival (and which actually may be detrimental) often at the expense of natural reward stimuli, is a clear example of the negative impact of emotional influence over cognitive systems. The degree of drug attentional bias has been shown to be related to craving levels (Franken et al., 2000; Field et al., 2009), such that greater bias is associated with enhanced drug craving. Furthermore, enhanced attentional bias after drug treatment predicts relapse to drug use in heroin (Marissen et al., 2006) and alcohol-dependent individuals (Garland et al., 2012). Thus, emotional biasing of cognitive processing appears to have a profound negative effect on clinical outcome.

## Anxiety and stress sensitivity in substance dependence

Whilst reward sensitivity is a long-established focus of substance dependence research, the contribution of changes within the brain's stress system is more recently being recognized as an important mechanism for the maintenance of addiction and also relapse to drug use during abstinence (Zhang et al., 2011).

Anxiety has been associated with substance use as a form of self-medication (Woicik et al., 2009) as alcohol and opiates have anxiolytic properties (Lejuez et al., 2006; Gilman et al., 2008; Colasanti et al., 2011). Anxiety is thought to reflect functions of a defence system that is activated by aversive, novel, and innate fear stimuli (Barros-Loscertales et al., 2006). In addition to threat stimuli, anxiety may also be produced by cognitive processes involved in the anticipation, interpretation, or recollection of perceived stressors or threats (Charney and Drevets, 2002).

The amygdala is critical in generating a response to such threat stimuli (LeDoux, 2007). Structures involved in anxiety that work in concert with the amygdala include other medial temporal structures, sensory cortices and thalamus, insula, hypothalamus, brain stem, and medial prefrontal cortex. The bed nucleus of the stria terminalis (BNST) mediates anxiety during exposure to less well-defined threatening environments or contexts that occur over several minutes (Charney and Drevets, 2002).

### The “anti-reward” system and substance dependence

**Figure 3:**
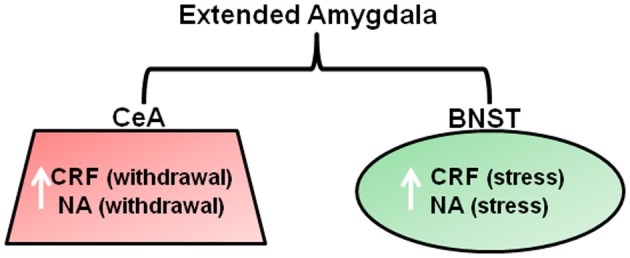
**Neurotransmitter changes within the brain stress system that are associated with withdrawal and stress-induced reinstatement of drug-seeking.** Neurochemical changes are proposed to occur within the extended amygdala in order to overcome the chronic presence of drugs of abuse. Withdrawal from all major drugs of abuse is associated with CRF and NA release within the CeA that produces anxiety like responses that are thought to drive drug seeking via negative reinforcement mechanisms. CRF and noradrenaline release within the BNST is considered to be important for mediating drug seeking in response to a stressor, such as a footshock (Koob, 2008). Abbreviations: CRF, Corticotrophic releasing factor; NA, Noradrenaline; CeA, central nucleus of the amygdala; BNST, bed nucleus of the stria terminalis.

As reviewed above, chronic drug use results in changes in reward systems leading to anhedonic states in some substance dependent individuals. Koob and colleagues additionally propose changes in “arousal-stress” systems during chronic drug administration, which are recruited in an attempt to overcome the presence of the drug and restore normal functioning (Koob, 2009). These systems include the hypothalamic pituitary axis (HPA) and extended amygdala (comprising the central nucleus, BNST, and a sub region of the nucleus accumbens). The extended amygdala receives afferent inputs from the basolateral amygdala and hippocampus and sends efferents to ventral pallidum and hypothalamus. Thus, it is ideally placed for its hypothesized role in opposing the rewarding effects of drugs of abuse, and has been referred to as the “anti-reward” system. Chronic drug administration involves the dysregulation of stress/anti-reward systems and neurochemical changes in the extended amygdala associated with arousal/stress modulation (Figure 3) that are associated with the emergence of negative emotional states such as anxiety and mood disturbances. Preclinical studies have demonstrated that chronic administration of all major drugs of abuse is associated with a release of corticotropic releasing factor (CRF) within the extended amygdala upon withdrawal and after stress induction with a footshock, that produces anxiety-like effects and drug-seeking that are reversed by CRF antagonists (Koob, 2008). These changes persist into protracted abstinence and are thought to contribute to relapse to drug-seeking in order to reduce negative emotional states (Kreek and Koob, 1998). Noradrenergic transmission within the extended amygdala has been associated enhanced anxiety and increased drug-seeking and relapse during abstinence in alcohol, cocaine, and opiate dependence (Koob, 2008; Smith and Aston-Jones, 2008). Furthermore, administration of lofexidine, a drug that reduces noradrenaline release, reduces stress and craving and improves abstinence in opiate users (Sinha et al., 2007). Seemingly contradictory to theories of heightened stress sensitivity in substance dependence, studies have demonstrated an apparent *insensitivity* to aversive stimuli in rats after extended cocaine self-administration (Deroche-Gamonet et al., 2004; Vanderschuren and Everitt, 2004). In these studies, the presentation of a CS that predicted an aversive event did not prevent responding for cocaine. However, the inability of the aversive CS to modulate behavior was attributed to the behavior in question being controlled by habit action selection systems (see later section entitled “Systems Involved in Action Selection”) that by their very nature, are immune to immediate changes to action outcomes.

### Human studies of anxiety in drug dependence

This hypersensitivity of the brain's anxiety/stress system is evident in clinical populations as opiate-dependents find unpleasant stimuli more arousing that controls (Aguilar de Arcos et al., 2005, 2008). Enhanced stress reactivity is also apparent in physiological measures of stress, such as systolic blood pressure (Sinha et al., 2008), cortisol response (Fatseas et al., 2011), and HPA response (Fox et al., 2005). For example, alcohol-dependent participants showed increased heart rate and cortisol levels compared to social drinking controls in response to stressful and alcohol-related images (Sinha et al., 2008).

Sensitized responses to stress are apparent in brain imaging studies. The extended amygdala and corticostriatal circuitry is involved in both reward and affective processing. Medial prefrontal cortex, anterior and posterior cingulate, striatum, and insula are associated with stress- and drug-cue-induced craving, which in turn are associated with increased susceptibility to relapse (Sinha and Li, 2007). Stress induction, using guided imagery of personal stressful and neutral situations in cocaine-dependent individuals and controls, resulted in increased response within the dorsal striatum that correlated with increased craving. Patients additionally demonstrated reduced activation in anterior cingulate and prefrontal regions compared with controls (Sinha et al., 2005). Using the same stress induction method, and drug cue exposure, a later study demonstrated that guanfacine, a α2 adrenoceptor agonist, increased prefrontal activity in response to induced stress and drug cue exposure, and reduced craving (Fox et al., 2012). A study in recently detoxified alcoholic-dependent individuals demonstrated that an NK-1 receptor antagonist reduced brain response to negative images in the inferior temporal gyrus, insula, and middle temporal gyrus and reduced serum cortisol levels and alcohol cue-induced cravings (George et al., 2008).

Anxiety/stress sensitivity is considered to maintain addiction (Heilig and Koob, 2007), and increase susceptibility to relapse during abstinence (Sinha, 2001; Duncan et al., 2007) in heroin (Fatseas et al., 2011), alcohol (Sinha et al., 2008), and cocaine-dependents (Karlsgodt et al., 2003).

## Impact of emotional processes on cognition in addiction

These dysfunctions influence the behavior of addicted individuals, tending to increase, and maintain drug-taking. In particular, emotional dysregulation and altered reward sensitivity may underpin impulsive behavior and poor decision-making. Both of these tendencies can be seen in the “real-world” behavior of addicted individuals, but can also be studied using laboratory-based paradigms.

### Affective impulsivity and substance misuse

As has been outlined, drug dependence is associated with a relative enhancement of processing of drug-related stimuli at the expense of natural rewards. This attentional bias is associated with the emotional state of craving and impacts upon relapse vulnerability. Furthermore, changes occur in “anti-reward” systems that result in negative emotional states maintain addiction via negative reinforcement mechanisms. However, addiction is associated with a loss of control over drug use which continues in spite of individuals' awareness of serious negative consequences. Increased reward and anxiety sensitivity alone do not seem a sufficient explanation for this persistent maladaptive behavior. Instead there must be additional deficits in decision-making and/or inhibiting maladaptive behaviors. These deficits may be mediated by reward and anxiety sensitivity, but critically involve these emotional factors exerting a detrimental effect on cognitive function. The term “impulsivity” is often used to describe behavior characterized by excessive approach with an additional failure of effective inhibition (Hommer et al., 2011) and has consistently been found to be associated with substance dependence (de Wit, 2009; Dalley et al., 2011). Impulsivity is a complex multifaceted construct which has resulted in numerous additional definitions such as, “the tendency to react rapidly or in unplanned ways to internal or external stimuli without proper regard for negative consequences or inherent risks” (Shin et al., 2012), or “the tendency to engage in inappropriate or maladaptive behaviors” (de Wit, 2009).

These definitions reflect different types of impulsivity. Examples include reflection impulsivity (action without adequate evaluation of the situation), impulsive action (inadequate motor inhibition), risky decision-making (impulsive choices of immediate rewards over larger delayed ones) (Dalley et al., 2011), and attentional impulsivity, or lack of perseverance (inability to focus on a task or goal) (Cyders and Smith, 2008). In addition, the recently defined constructs of positive and negative urgency reflect the tendency to act rashly in response to extreme negative or positive affect (Cyders and Smith, 2008). Whilst these varieties of impulsivity involve different psychological processes, it is likely that they interact to modulate behavior (Evenden, 1999).

#### Questionnaire measures of emotional impulsivity

Self-report questionnaires are frequently used to assess impulsivity. Distinctions have been made between measures of cognitive impulsivity (reflection impulsivity, attentional impulsivity) and emotional impulsivity (positive and negative urgency) (Fernandez-Serrano et al., 2012).

Deficits in cognitive impulsivity have been identified across alcohol (Evren et al., 2012), heroin (Nielsen et al., 2012), and cocaine addiction (Ersche et al., 2010b) using measures such as the Barratt Impulsivity Scale (BIS-11) with higher scores predicting greater drug use (Ersche et al., 2010b) and relapse (Evren et al., 2012). Longitudinal studies have demonstrated that impairments in emotional and behavioral regulation confer a risk for the later development of substance abuse. The trait of behavioral disinhibition in young adults, which reflects impulsive novelty-seeking, was found to predict substance abuse 6 years later (Sher et al., 2000). The construct of neurobehavioral disinhibition is indexed by self-report measures of emotional regulation, parent and teacher indicated measures of behavioral control, and performance on tests of executive functioning. Neurobehavioral disinhibition in 10–12 years old has been shown to be consistent in predicting later development of substance abuse in young adulthood (Tarter et al., 2003; Kirisci et al., 2007).

Thus, cognitive impulsivity appears to be associated with addiction, and may play a role in the development of substance misuse. However, in this review, we will focus on emotional impulsivity, which more closely reflects the interaction between emotional and cognitive processes. Impulsivity defined as “the inability to control behavior in the face of reward and/or punishment” is associated with increased substance use in young adults. Both positive and negative reinforcement motives are associated with this impulsivity trait (Woicik et al., 2009), suggesting that increased substance use may be related to an inability to control behavior when experiencing either positive or negative emotion. Both negative and positive urgency were found to be higher in polysubstance users. Positive urgency scores correlated with amount of cocaine use and binge drinking, whilst scores on measures of reflection impulsivity did not differ from controls (Verdejo-Garcia et al., 2010). In addition, both positive and negative urgency have been shown to be correlated with problem drinking in undergraduate students (Cyders et al., 2007), and to differentiate substance abusers from controls (Cyders et al., 2007). In a study investigating impulsivity dimensions, higher scores on measures of reflection impulsivity, attentional impulsivity, and negative urgency all differentiated substance dependents from controls, although negative urgency was found to be the best predictor of alcohol, drug, social, legal, medical, and employment problems (Verdejo-Garcia et al., 2007a).

Although impulsivity is a multifaceted construct, comprising different psychological processes, failings across all dimensions of impulse control occur in substance dependence. However, findings above highlight a specific role for emotion, both positive and negative, in producing impulsive behaviors. Emotional impulsivity traits appear distinct from other impulsivity traits and particularly pertinent for dependence, reliably differentiating substance users from controls, and also predicting poorer outcomes in dependent individuals.

#### Behavioral measures of affective impulsivity

Self-report measures rely upon the accuracy of the individual's introspection. Behavioral measures offer an index of impulsivity that is free of subject bias. There are two broad categories of behavioral impulsivity measures. One is characterized by deficits in the ability to inhibit a motor response, referred to as behavioral inhibition. The other is associated with a deficit in inhibition that is motivationally driven and is associated with reward processing (Castellanos-Ryan et al., 2011). Deficits in behavioral inhibition have been found in substance dependence (Forman et al., 2004; Hester and Garavan, 2004; Noel et al., 2007; Fu et al., 2008), consistent with the role of cognitive impulsivity in addiction. However, here we will focus on reward-based impulsivity, reflecting the impact of emotional processing on cognitive performance.

A common behavioral measure of impulsivity is the delay discounting task which measures the degree of temporal discounting. Temporal discounting describes the process by which the subjective value of a reward decreases as a function of delay to that reward (Bickel et al., 2007). Participants are faced with the choice of a small immediate reward, or a larger delayed reward; choosing the smaller immediate reward indicates a higher degree of impulsivity. Increased discounting of larger delayed rewards has been found in heroin- (Madden et al., 1997; Kirby et al., 1999; Kirby and Petry, 2004), cocaine- (Coffey et al., 2003; Kirby and Petry, 2004), and alcohol (Petry, 2001; Bjork et al., 2004a; Mitchell et al., 2007) -dependent individuals. Drug rewards are discounted at an even higher rate than monetary rewards (Madden et al., 1997, 1999; Kirby et al., 1999). Enhanced discounting is also seen during mild opiate withdrawal, possibly reflecting the emergence of negative affect states during withdrawal (Koob and Le Moal, 2005). There is evidence that delayed discounting is influenced by emotional state in healthy controls, with positive mood induction increasing discounting of larger delayed rewards in extraverted individuals (Hirsh et al., 2010). This effect, reflecting a complex interaction between reward sensitivity, emotional state, and cognition, does not appear to have been tested in drug users, although it is an obvious area for study, given that all three intersecting factors are abnormal in addiction.

Emotional influences on decision-making can be measured empirically, using tasks where higher level cognitive processing is regulated by emotion and feeling (Bechara, 2005). The Iowa Gambling Task was developed to test “emotional” decision-making in a laboratory setting for patients with ventromedial prefrontal cortex damage (Bechara et al., 1994). This task presents choices between large monetary gains (but with associated even larger losses, such that the overall long-term outcome is loss) and small monetary gains (but with associated smaller losses, such that the overall long term outcome is gain) (Bechara et al., 1994). Impairments in this task, in the form of disadvantageous choices despite rising losses, have been found in cocaine (Stout et al., 2004; Verdejo-Garcia et al., 2007b; Cunha et al., 2011), heroin (Petry et al., 1998; Verdejo-Garcia et al., 2007b), and alcohol addiction (Bechara et al., 2001; Noel et al., 2007). In the Iowa Gambling Task, reward outcome probabilities are unknown, therefore participants have to learn reward contingencies. This places high demands on “cold” executive processing as well as “hot” emotional processing that may bias decision-making toward high rewards in spite of the negative consequences. The task thus provides an ideal test of how emotional processing impacts upon cold cognition, but does not dissociate the contribution of affective and cognitive processes to any deficits. The Cambridge Gamble (Rogers et al., 1999a) and the Cambridge Risk Task (Rogers et al., 1999b) require less learning and working memory processing, as outcome probabilities are presented explicitly. Studies with the Cambridge tasks also find deficits in opiate (Rogers et al., 1999a; Fishbein et al., 2007; Passetti et al., 2008), stimulant (Rogers et al., 1999a,b), and alcohol-dependent subjects (Bowden-Jones et al., 2005; Lawrence et al., 2009). Furthermore, poorer decision-making confers a greater risk of relapse in opiate- (Passetti et al., 2008) and alcohol (Bowden-Jones et al., 2005) -dependent individuals.

Bechara et al. demonstrated an enhanced affective response to anticipated and actual gains during the IGT in substance dependent individuals in the form of elevated skin conductance, and a reduced skin conductance response before making a risky decision (Bechara et al., 2002; Bechara and Damasio, 2002). They concluded that hypersensitivity to reward and an impaired ability to use affective signals to guide behavior, underlie impaired decision-making in these individuals. In support of reward hypersensitivity underlying IGT deficits in substance abusers, measures of novelty-seeking have been found to predict poor IGT performance in alcohol-dependent subjects (Noel et al., 2011). Note that hypersensitivity to rewards in this context is somewhat at odds with the findings from the monetary incentive delay task reported earlier. Money can be considered to be a drug cue (Garavan et al., 2000), as it is necessary for obtaining drugs, however, only when presented in sufficient quantities. Gambling tasks typically involve presentations of much larger and more salient sums than the monetary incentive delay task.

Impaired decision-making in the face of motivationally salient outcomes is a core deficit in addiction, with individuals opting for immediate rewards, despite negative longer-term outcomes. Substance dependence involves the choice of immediate drug reward despite negative long term consequences (e.g., health, family, economic, and criminal problems) and these deficits thus provide an extremely plausible model of how motivational factors negatively influence real world decision-making.

#### Studies demonstrate the impact of emotional state on decision-making

Specifically assessing the influence of emotional processing on decision-making, studies in healthy volunteers have demonstrated that high levels of trait anxiety (Miu et al., 2008), negative affect (Suhr and Tsanadis, 2007), sensation-seeking (van Honk et al., 2002; Suhr and Tsanadis, 2007), and stress sensitivity (van den Bos et al., 2009) are predictive of poor decision-making on the IGT.

High levels of negative affect, anxiety/stress sensitivity and sensation-seeking in substance dependent individuals may therefore contribute to observed deficits on decision-making tasks. Reward and stress mechanisms are considered to be important mechanisms underlying relapse (Stewart, 2008), suggesting these emotional traits impair real life decision-making. Studies directly assessing the role of emotional states on decision-making in opiate addiction have shown that trait and state anxiety are negatively correlated with performance on the IGT (Lemenager et al., 2011). Furthermore, stress induction using the Trier Social Stress Test, was shown to produce a significant deterioration in IGT performance in long term abstinence and newly abstinent heroin users, but not in comparison subjects. Treatment with the B adrenocepter antagonist propranolol blocked the deleterious effect of stress on IGT performance, supporting the role of the noradrenergic system in the generation of negative emotional states in substance dependence (Zhang et al., 2011). A later study from the same group found that drug cue exposure increased craving and impaired performance of the IGT in long term and newly abstinent heroin users (Wang et al., 2012), indicating that conditioned emotional responses impair decision-making. Interestingly, in a group of heavy drinkers, the induction of anticipatory stress (by making participants believe they were required to carry out an embarrassing speech) before the IGT task *improved* performance of the IGT. This effect was attributed to a greater sensitivity to losses after stress induction (Gullo and Stieger, 2011). Similarly, the induction of negative affect via exposure to negative images from IAPS, improved performance on the IGT task in cocaine-dependent participants (Fernandez-Serrano et al., 2011). These latter studies suggest stress induction can have bivalent effects on decision-making.

The reviewed studies demonstrate that decision-making is influenced by both trait and state affective processes in addiction. A deleterious effect of both trait and state anxiety was found in opiate addiction, although the induction of negative affect states improved performance in heavy alcohol drinkers and cocaine-dependent subjects. The tendency of stress in opiate users to bias decision-making in the favor of immediate rewards at the expense of long term goals is consistent with the finding that enhanced stress reactivity increases relapse susceptibility (Karlsgodt et al., 2003; Sinha et al., 2008; Fatseas et al., 2011). By contrast, stress induced enhancement of decision-making in cocaine and alcohol-dependent individuals seems at odds with the predicted roles of stress in addiction (Koob, 2008), and warrants further study with tasks designed to probe specific sub-processes.

One explanation for these contradictory findings may be the notion that stress can have differential effects on decision-making depending upon the degree of stress experienced. The stress response is an adaptive response to enable organisms to adequately deal with threats within the environment, however, an excessive or unduly persistent stress response can be detrimental (McEwen, 2007). It has been suggested that a certain levels of stress can be optimum for decision-making—according to the somatic marker hypothesis (Damasio, 1994) (see section “Influence of Somatic Markers” for a detailed description of this hypothesis), emotions can facilitate decision-making by rapidly signaling the prospective consequences of an action and accordingly assists the selection of the most advantageous response (Bechara and Damasio, 2005). Anxiety is considered to increase arousal and sensitivity to stimuli signaling punishment (McNaughton and Corr, 2004). Gullo and Stieger (2011) demonstrated that stress induction increased attention toward losses in heavy drinkers. Induced moderate stress/negative affect may improve IGT performance by enhancing punishment sensitivity (Fernandez-Serrano et al., 2011). More intense emotion however, may have a deleterious effect on decision-making. It has been suggested that excessive stress, anxiety and worry require emotional regulation, which may tax cognitive resources (Tice et al., 2001) and therefore impair performance on decision-making tasks (Miu et al., 2008). High levels of stress may produce a high level of “background” emotion that “drowns out” affective signals during performance of decision-making tasks (Gullo and Stieger, 2011).

A related explanation for the different findings of the impact of stress on decision-making may be different methods used for stress induction resulting in different levels of stress in each study. Zhang et al. induced stress using the Trier Social Stress Test, where participants are asked to carry out a short presentation and then perform difficult mathematical subtractions, all whilst being filmed. Gullo and Stieger induced stress by *informing* participants they would have to carry out an embarrassing presentation, although the participants did not actually do so. Fernandez-Serrano et al. induced negative affect by viewing negative and aversive pictures. It therefore could be argued that the levels of stress induced by the actual carrying out of the stressful procedures of the Trier Social Stress Test were greater than those induced by the anticipation of carrying out stressful procedures or by viewing negative pictures.

**Figure 4:**
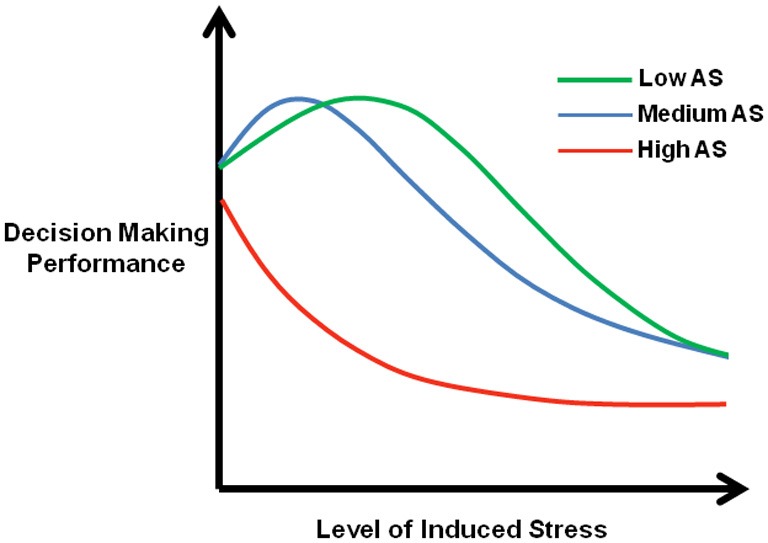
**Hypothetical relationship of the effect of stress on cognitive performance in substance dependence according to different trait levels of stress sensitivity.** Mild stress improves performance in those of low and medium AS traits by enhancing arousal and sensitivity to punishment signals. Increasing stress results in an increasing need for affect regulation, thus resulting in reduced task-related cognitive resource allocation and impaired performance. High trait AS is associated with impaired decision-making performance before stress induction (Lemenager et al., 2011) and thus further increases in stress are likely to lead to further significant task impairments. Abbreviations: AS, Anxiety Sensitivity.

Another factor that may influence task performance may be due to differences in study populations. Lemenager and Zhang studied opiate-dependent individuals, whereas Gullo and Steiger and Fernandez-Serrano studied a group of heavy drinking undergraduate students (who were drinking harmful levels of alcohol) and cocaine-dependent subjects respectively. Anxiety sensitive traits have been shown to be more specifically associated with heroin dependence than cocaine use (Lejuez et al., 2006). Whilst anxiety sensitive traits have been associated with alcohol dependence (Norton et al., 1997), this trait was not found to be associated with heavy drinking in young adults, rather, heavy drinking was more associated with sensation-seeking in this sample (Woicik et al., 2009). It is therefore likely that baseline anxiety levels and susceptibility to stress were higher in the studies of Lemengaer and Zhang than those of Fernandez-Serrano et al., and Gullo and Stieger. Therefore, different procedures and different populations (Figure 4) may combine to account for discrepancies in this literature.

### The impact of emotion on impulsive action and decision-making

The preceding sections have reviewed evidence of emotional disturbances and impaired decision-making in substance dependence. Decision-making involves processes of deciding upon the most appropriate actions to take after consideration of the predicted value of an action (Redish et al., 2008), and the automatic selection of actions that an agent has learnt, through past experience, delivers high valued outcomes. The predicted value of an action can be defined in terms of the expected reward it is expected to elicit (after consideration of the probability of, and delay to, receiving the reward), minus any costs associated with the action (Daw et al., 2005). The following sections will review how decision-making can be modulated by emotional processes, and how dysregulation of these processes in substance dependence contributes to decision-making deficits.

#### Systems involved in action selection

Primate and rodent studies suggest systems controlling behavior can be separated into planning and habit systems. Planning systems (also referred to as deliberative, cognitive, reflective or executive systems) are “goal-directed” systems that allow an agent to consider the possible consequences or outcomes of its actions to guide behavior. Habit systems mediate behaviors that are triggered in response to certain stimuli or situations but without consideration of the consequences. Given the differential consideration of action outcomes, behaviors controlled by the planning system are sensitive to outcome devaluation, whereas habitual behaviors are not (Niv et al., 2006; Redish et al., 2008). In addition, emotional circuit activation in response to a biologically significant event, or the cues that predict it, can elicit a series of evolutionary hard-wired Pavlovian actions such as approach, freezing and fleeing arising from an expectancy generated by the CS-UCS association. Brain areas underlying Pavlovian responses include the amygdala, which identifies the emotional significance or value of external stimuli, and the ventral striatum, which mediates motivational influences on instrumental responding (Cardinal et al., 2002), and their connections to motor circuits (van der Meer et al., 2012). Thus, it has been argued that emotions constitute a decision-making system in their own right, exerting a dominant effect on choice in situations of opportunity or threat (Seymour and Dolan, 2008; van der Meer et al., 2012).

There are computational differences in how the habitual and planning systems decide upon appropriate actions to take. The process of action selection in the planning system is complex. It involves searching through and predicting the possible consequences of actions. Consequences are evaluated online, taking current needs and motivational state, time, effort, and probability of receiving the desired outcome into consideration before a decision is made (Redish et al., 2008; van der Meer et al., 2012). Due to the numerous searches of different actions and their potential consequences, this process is slow, requiring extensive cognitive processing. Action selection processes are flexible, allowing an organism adapt behavior to changing environments and needs. The habit system chooses actions based upon stored associations of their values from past experience; through training, an organism learns the best action to take in a certain situation. Upon recognition of the situation again this “best action” will automatically be initiated, without consideration of consequences of such an action. This process is very fast but inflexible, unable to adapt quickly to changes in the value of outcomes (Daw et al., 2005; Redish et al., 2008). Under appropriate conditions, habitual actions may be overridden by planning systems (Redish et al., 2008).

#### The influence of emotional processes on action selection

Emotional processing involves detecting and responding to salient challenges and opportunities within the environment to enable an organism to thrive and survive. Key brain circuits involved in these processes are the reward and stress circuitry that are involved in reinforcement, motivation, and defence. In the following section we will argue that brain emotional systems have a key role in decision-making.

***Influence of emotion in the planning system: neural substrates.*** As described, the planning system selects actions after consideration of potential outcomes, and is sensitive to changes in outcome value. Therefore, the planning system will be influenced by brain areas involved in evaluating and predicting outcome values (Redish et al., 2008; Balleine and O'Doherty, 2010). The OFC of the planning system has been demonstrated to be involved in the valuation of reward outcome (Elliott et al., 2003) and the predictive value of CS (Tremblay and Schultz, 1999), therefore emotional processing by the OFC is integral to the planning system. The OFC additionally works in concert with subcortical emotional systems in the valuation of outcomes; the amygdala is considered to be a key neural substrate for outcome valuation due to its sensory and hypothalamic afferents which allow for the integration of specific sensory features of outcomes with emotional feedback (Balleine and O'Doherty, 2010). The basolateral amygdala also mediates the influence of CS on goal directed behavior by allowing a CS to access the current value of the UCS that it predicts (Seymour and Dolan, 2008; Cardinal et al., 2002). Basolateral amygdala lesions rendered the instrumental performance of rats insensitive to outcome devaluation (Balleine et al., 2003) and blockade of opioid receptor signaling in the basolateral amygdala with naloxone prevented the effect of food deprivation to increase food-seeking (Wassum et al., 2009). Both of these results suggest that disrupting basolateral amygdala functions prevents the use of new outcome values to guide behavior within the planning system. Other neural structures assumed to be important in the valuation of outcomes include the ventral pallidum, encoding the hedonic impact of rewards (Tindell et al., 2004), and the ventral striatum that appears to have a role in encoding reward value (Schultz et al., 1997; McDannald et al., 2011).

Pavlovian CS can influence instrumental performance by a process known Pavlovian-instrumental-transfer (PIT). A reward associated CS can enhance instrumental responding specifically for the reward that it associated with (outcome specific PIT) or it may enhance responding generally by enhancing arousal (general PIT) (Corbit and Balleine, 2011). PIT is considered to reflect an effect of increased incentive motivation to increase response vigor (Everitt and Robbins, 2005). PIT depends upon the amygdala and nucleus accumbens, with the latter thought to have a role in translating motivation into action (Cardinal et al., 2002; Balleine and O'Doherty, 2010).

***Influence of emotion within the habit system.*** Behaviors controlled by the habit system are carried out without consideration of outcome value, but instead reflect automatic responses to a stimulus or situation. As we have seen, emotional processing appears to have an important role in integrating homeostatic needs in the calculation of outcome values, creating incentive motivation toward highly values outcomes. It might therefore be expected that incentive motivation would not affect habitual behaviors that act independently of outcome value. However, this is not the case: shifts in primary motivation (e.g., from hunger to satiety) have been shown to affect the vigor of habitual actions. This is thought to be due to a “generalized drive” effect that is distinct from specific outcomes (Niv et al., 2006), suggesting there is a general activating effect of motivation state. Habitual actions are additionally influenced by Pavlovian predictors of rewards (Balleine and O'Doherty, 2010)—overtraining actions that increase insensitivity to outcome value (i.e., reflecting a transition from planning system to habit system control) render actions more sensitive to appetitive CS effects on response vigor (Belin et al., 2009). Furthermore, it has been demonstrated that a CS continues to influence instrumental actions that result in the outcome it predicts, even after the earlier devaluation of the outcome (Rescorla, 1994), suggesting both general PIT and outcome specific PIT effects influence habitual actions. Thus, whilst outcome values do not affect habitual actions, general shifts in motivation and Pavlovian cue values, encoded by the ventral striatum and OFC, do in a manner that is independent of representation of current outcome value (Balleine and O'Doherty, 2010).

***Influence of somatic markers.*** An important component of the emotional response is changes within the internal milieu and viscera of the body such as a release of hormones and increase in heart rate (Bechara and Damasio, 2005). These physiological changes within the body are relayed back to the brain with areas such as the insula and the somatosensory cortices suggested to convert these physiological signals into subjective feeling states (Damasio, 1994). Damasio's Somatic Marker Hypothesis highlights the specific role of these physiological signals arising from the body in the guidance of behavior (Damasio, 1994). The term “somatic state” is used to describe the brain and body responses to UCS and CS (referred to as “primary inducers”). Once a somatic state has been triggered by a primary inducer and experienced at least once, a representation of this somatic state is formed. The theory proposes that somatic state can be “reactivated” by thoughts and memories of real or imagined emotional events (referred to as secondary inducers'). This reactivation of somatic states by secondary inducers is proposed to guide future decisions by signaling the prospective consequences of an action.

Brain areas important for generating somatic states from primary inducers include the amygdala and effector structures such as the hypothalamus, autonomic brain stem nuclei, the ventral striatum and periacqueductal grey (PAG). The medial orbitofrontal/ventromedial prefrontal cortex (defined as VMPFC) is responsible for triggering somatic states from secondary inducers. It is proposed the VMPFC couples recalled or imagined scenarios (supported by the hippocampus and DLPFC) with brain areas important for the representation of somatic states within the insula, somatosensory cortices, posterior cingulate/precuneus region. Somatic states may influence decision-making with or without conscious knowledge in the striatum and in the prefrontal cortex respectively (Verdejo-Garcia and Bechara, 2009).

***Distracting effect of emotions.*** Emotion can guide decision-making when it is integral to the task at hand, emotional responses that are unrelated, or excessive, can be detrimental (Bechara and Damasio, 2005). Dorsal prefrontal regions are involved in the regulation of affective states (Phillips et al., 2003a). Excessive emotion is likely to require regulation by these areas (Phillips et al., 2003b; Amat et al., 2005; Robbins, 2005). Dorsal prefrontal regions are additionally important in decision-making and inhibitory control, thus high levels of emotion that require regulation may limit resources available for these functions, which may contribute to deficits in decision-making.

#### The effects of emotional processing in substance dependence

Given the crucial role of emotions in the processes of decision-making as described above, along with evidence that both craving and stress are significant drivers of relapse (Weiss, 2005; Sinha, 2007), it follows that dysregulation of emotional processing may contribute to the observed decision-making deficits observed in substance dependent individuals.

***Effects within the planning system.*** As reviewed, the brain's reward system has a heightened sensitivity to conditioned drug stimuli. Various mechanisms are proposed to underlie this effect including pre-morbid vulnerabilities in brain emotional systems and the action of drugs of abuse within brain emotional systems. One mechanism is drug-induced sensitization of dopamine neurons that may act to enhance the incentive salience of drug cues, increasing “wanting” of drug outcomes (Robinson and Berridge, 1993). Given that CSs reflect the value of the reward that they predict (Seymour and Dolan, 2008), an additional explanation of the heightened response to drug cues is due to overvaluing of drugs of abuse within the reward system (Redish et al., 2008). This overvaluing has been attributed to an increased hedonic impact of drugs of abuse, via enhanced opioid receptor signaling (Berridge and Kringelbach, 2008; Redish et al., 2008). Pharmacological actions of drugs of abuse to increase dopamine release in the nucleus accumbens have also been suggested to mediate their pleasurable effects (Volkow et al., 2011). Therefore, dopamine mediated increases in hedonic impact may also contribute to overvaluation. However, the role of dopamine in drug “liking” has been questioned (Berridge, 2007). Drug induced dopamine release may also influence decision-making via effects on Pavlovian learning. Learning of Pavlovian values is mediated by the difference between what is expected after presentation of a CS, and the outcome actually received, referred to as a prediction error. This prediction error can either be positive, indicating a better outcome than expected or negative, indicating a worse outcome than expected (Balleine and O'Doherty, 2010). Prediction errors result in modification of the predictive outcome value assigned to a CS. Dopamine neuron firing appears to encode prediction errors (Schultz, 1998), suggesting phasic dopamine signaling may be the teaching signal that enables the learning of Pavlovian associations. Therefore, increases in phasic dopamine release produced by drugs of abuse, may signal a positive “better than expected” prediction error, resulting in an increase in the predictive outcome value assigned to drug cues (Redish et al., 2008). However, the role of dopamine in learning is still under debate, as it is suggested that dopamine neuron firing is not the teaching signal that causes learning, but instead is a consequence of learning that occurs elsewhere (Berridge, 2007).

Homeostatic and allostatic changes associated with chronic drug use may result in withdrawal symptoms, excessive anxiety sensitivity, and depressive symptoms. This may result in an overvaluing of drugs of abuse that satisfy a homeostatic need, such as immediate withdrawal, anxiety and depressive symptom relief (Redish et al., 2008; Verdejo-Garcia and Bechara, 2009). Increased drug use to alleviate negative emotional states is suggested to underlie the compulsive nature of drug use in substance dependence. Compulsive disorders are associated with anxiety and stress before committing a compulsive act, and relief from that stress by carrying out the act (Koob and Volkow, 2010). In addition, allostatic changes within the reward system appear to have the effect of undervaluing natural rewards, thus further biasing decision-making in favor of drug rewards.

As reviewed, the planning system guides decisions after considering the outcome value of actions, in the context of current needs. Therefore enhanced outcome values of drug use may bias decision-making systems in favor of drug use. The amygdala, which plays a key role in detecting the emotional significance of a CS and generating appropriate responses, mediates the influence of overvalued drug outcomes on drug-seeking behavior after exposure to drug-related stimuli (Bechara, 2005). According to the somatic marker hypothesis, the VMPFC is involved in guiding behavior in line with long term goals (such as of abstinence) by evoking somatic states from thoughts or memories.

In addition to being important for the generation of somatic states, prefrontal regions are also crucial for cognitive and motor inhibitory control (Aron, 2007). A consistent finding of neuroimaging studies of decision-making in substance dependence is hypoactivation of the prefrontal cortex (Bolla et al., 2003; Tanabe et al., 2007; Bjork et al., 2008b), although hyperactivation in the lateral OFC has also been found in opiate and amphetamine-dependent individuals (Ersche et al., 2005). Chronic drug use is consistently associated with VPFC, DLPFC and ACC gray matter loss in cocaine and alcohol dependence (Fein et al., 2002a,b; Makris et al., 2008; Fein et al., 2010; Goldstein and Volkow, 2011; Ersche et al., 2011) and reduced prefrontal neuronal viability in opiate dependence (Haselhorst et al., 2002; Yucel et al., 2007). VPFC and DLPFC loss have been shown to predict both impaired performance on the IGT (Tanabe et al., 2009) and preference for immediate gratification in delay discounting tasks (Bjork et al., 2009) Such findings suggest that the prefrontal regions of the planning system is impaired in substance dependence, compromising both the ability to generate affective states relating to long term goals (Bechara and Damasio, 2005) and the ability to exert executive inhibitory control over drug-seeking thoughts and actions (Goldstein and Volkow, 2011).

Dorsal prefrontal regions are involved in the regulation of affective states (Phillips et al., 2003a). Therefore excessive anxiety or craving would require regulation by these areas. Studies have shown dorsal prefrontal regions to be important in regulating craving and reducing amygdala activity in cue induced craving paradigms (Brody et al., 2007; Kober et al., 2010). Considering these prefrontal regions are important for decision-making, craving and anxiety regulation would limit the resources available for effective decision-making within the planning system.

***Effects within the habit system.*** A transition from control over drug-seeking by planning systems to control by habit systems has been proposed to underlie compulsive drug use (Everitt and Robbins, 2005). As reviewed, Pavlovian cues have motivational impacts upon habitual actions in a manner that is independent of representation of current outcome values (Balleine and O'Doherty, 2010). Therefore, drug cues have the ability to increase drug-seeking actions controlled by habit systems. This transition to habitual control in substance dependence means drug-associated cues come to enhance drug-seeking, via PIT mechanisms, without consideration of the consequences of drug-seeking actions.

Preclinical studies have demonstrated that the transition from goal-directed to habitual behavior is associated with a change from dopamine release in the ventral striatum to the dorsal striatum in response to drug associated stimuli (Everitt et al., 2008). PET studies have demonstrated cue induced dopamine release to occur in the dorsal, but not ventral, striatum of cocaine (Volkow et al., 2006) and heroin-dependent individuals (Zijlstra et al., 2008), providing evidence that habitual system control may underlie compulsive drug-seeking in these dependent groups.

The mechanism of this transition to dorsal striatal control appears to be a dopamine-dependent mechanism, and thus drug-induced sensitization of dopamine neurons, and pharmacological action of drugs of abuse to increase striatal dopamine release, may accelerate this transition (Everitt et al., 2008). Prediction error dopamine neurons innervate the dorsal as well as the ventral striatum (Everitt and Robbins, 2005) and thus drug induced positive prediction errors may result in overvaluing of actions that lead to drug use, enhancing the consolidation of these stimulus-response relationships (Everitt et al., 2008; Redish et al., 2008). Furthermore, recent evidence suggests a role of stress in shifting goal-directed control to habitual control of behavior (Schwabe and Wolf, 2011). This effect appears to be mediated by the action of both cortisol and noradrenaline (Schwabe et al., 2010). Therefore sensitization of the brain's stress system occurring with chronic drug use is likely to contribute to the development of habitual drug-seeking.

A shift in the control of drug-seeking to habitual system control is disadvantageous, potentially reflecting a “loss of control” over drug-seeking that is insensitive to devaluation of drug rewards. This may explain the persistence of drug use despite explicit knowledge of negative consequences, which under goal-directed control, should reduce the propensity for drug-seeking. Whilst there is evidence that automatic, habitual actions may be overridden by controlled, planning-like systems (Redish et al., 2008), impaired function of prefrontal planning system regions observed in substance dependence suggest the ability to regain control of drug-seeking may be compromised in substance dependent individuals.

## Summary and conclusions

It is clear that substance dependence is associated with significant emotional dysregulation that influences cognition via numerous mechanisms. This dysregulation comes in the form of heightened reward sensitivity to drug-related stimuli, reduced sensitivity to natural reward stimuli, and heightened sensitivity of the brain's stress systems that respond to threats. Such disturbances have the effect of biasing attentional processing toward drugs with powerful rewarding and/or anxiolytic effects at the expense of natural rewards, resulting in profound negative effects on clinical outcome. Emotion dysregulation can also result in impulsive actions and influence decision-making, via a range of mechanisms. Overvaluing of drug rewards may enhance goal directed drug-seeking behaviors controlled by planning systems. Furthermore, learned Pavlovian values associated with drug stimuli motivate drug-seeking controlled by habit systems, resulting in automatic drug-seeking when exposed to drug cues and environments. Actions of drugs within brain reward and stress systems may additionally accelerate the transition from planning system to habit system control over drug-seeking. Such accelerations of this transition are extremely detrimental clinically, as drug-seeking behaviors under habit system control are impulsive, initiated without consideration of potential negative consequences of drug use. This has implications for treatment of substance dependence. Psychological therapies aimed at focusing upon the negative consequences of drug use or punitive measures aimed to reduce drug use, will be more effective when drug-seeking is under planning system control, but possibly ineffective for drug use that is under habitual control. Reducing exposure to drug associated cues and contexts is crucial in reducing habitual drug-seeking, although this may not be practical. An exciting area of research that may prove promising for the treatment of habitual drug-seeking is to effectively “wipe” drug memories via the combination of drug memory reconsolidation and extinction processes (Xue et al., 2012).

Impulsive drug-seeking and insensitivity to negative consequences are worsened by impairments of prefrontal systems that serve to generate “warning” somatic emotional signals when considering drug use. This result in the amygdala dominating somatic signaling, acting to incentivize drug-seeking by creating expectancies of large rewards after drug cue exposure. Extreme emotion requiring regulation from frontal brain regions, such as excessive anxiety associated with substance dependence, may further impair decision-making within the planning system by limiting available resources that can be allocated to assessing possible consequences of each action. There is likely to be significant variability in the extent to which these distinct, but inter-related mechanisms confer vulnerability to developing long-term addictions. This variability is influenced by differences in the pharmacological actions of the drugs abused, pre-morbid individual trait differences, and differences in the environments of drug users.

We have outlined evidence that emotional processing significantly impairs cognition in substance dependence. Emotionally influenced cognitive impairments have serious negative effects on clinical outcome, with both attentional bias and decision-making deficits being predictive of drug relapse. However, emotional processing has evolved to enable an organism to take advantage of opportunities, and effectively cope with challenges within the environment. The influence of emotion is clearly detrimental in substance dependence, and many of the detrimental effects observed are due to the ability of drugs of abuse to mimic the effects of stimuli or events that have survival significance. Drugs of abuse effectively trick the brain's emotional systems into thinking that they have survival significance, resulting in their high valuation and overvaluing of actions that lead to drug use. The biasing of cognition in favor of the procurement of highly valued substances is an entirely adaptive process. Unfortunately, for substance dependent individuals, the most highly valued substances (drugs) are devoid of positive survival significance, instead having a significant negative impact upon survival.

### Future directions

Although the current review focuses on drug dependence, the study of non-substance addictions may help improve understanding of addiction. Investigation of these maladaptive behaviors allows us to explore fundamental mechanisms of addiction, without the confounding neurotoxic effects of substance use (Bechara, 2003; Verdejo-Garcia et al., 2008). Pathological gambling and other so-called “behavioral addictions,” involving activities such as playing computer games, eating and shopping, appear to share some common mechanisms with substance dependence (Shaffer et al., 2004; Verdejo-Garcia et al., 2008). Behavioral addictions are associated with emotional and cognitive dysfunctions within the reward (Potenza, 2008; Balodis et al., 2012) and stress systems (Meyer et al., 2000; Moodie and Finnigan, 2005) as well as in impulsive and decision-making processes (Bechara, 2003) which negatively impact on outcome (Elman et al., 2010). Therefore further work on a generic “addiction endophenotype” is warranted.

It is also important to emphasize the need for future investigations into the differences between different substance dependences, particularly in relation to vulnerability to dependence. It is likely that drugs with different pharmacological properties have distinct actions on brain reward and stress systems. However, studies are typically confounded by polydrug use in the UK and other western countries. Studies of single drug addiction may be more feasible in countries where polydrug use is less prevalent.

Substance dependence research would also greatly benefit from longitudinal studies assessing factors conferring risk for initial substance use; how these factors relate to the subsequence development of dependence; how these factors change within the course of long term dependence; and how these factors impact upon outcome. It is likely that each “stage” of drug use is associated with specific cognitive mechanisms, but they are all likely to be characterized by dysregulated emotional processes influencing behavior and cognition. There is also a need for more sophisticated assessments of emotion processing and decision-making, particularly building on recent advances in understanding brain and behavioral mechanisms of socioeconomic decisions and social cognition.

Longitudinal studies will inform treatment development, particularly of therapies targeting prevention in individuals at high-risk. As we have emphasized in this review, once heavy drug use is initiated, changes within structures involved in reward, stress and executive functioning create a reinforcing cycle which is extremely difficult to break. However, treatment strategies based on emotion regulation and strengthening cognitive control processes in high-risk individuals could potentially prevent the cycle developing, and therefore holds enormous promise in preventing addiction and its many associated harms.

### Conflict of interest statement

The authors declare that the research was conducted in the absence of any commercial or financial relationships that could be construed as a potential conflict of interest.
